# Psychometric properties of the Patient-Reported Outcomes Measurement Information System (PROMIS®) pediatric item bank peer relationships in the Dutch general population

**DOI:** 10.1007/s11136-021-02781-w

**Published:** 2021-02-19

**Authors:** Michiel A. J. Luijten, Raphaële R. L. van Litsenburg, Caroline B. Terwee, Martha A. Grootenhuis, Lotte Haverman

**Affiliations:** 1 Child and Adolescent Psychiatry & Psychosocial Care, Amsterdam Reproduction and Development, Amsterdam Public Health, Emma Children’s Hospital, Amsterdam UMC, University of Amsterdam, Meibergdreef 9, Postbus 22660, 1100 AD Amsterdam, The Netherlands; 2 Department of Epidemiology and Data Science, Amsterdam Public Health Research Institute, Amsterdam UMC, Vrije Universiteit Amsterdam, de Boelelaan 1117, Amsterdam, The Netherlands; 3 Princess Máxima Center for Pediatric Oncology, Utrecht, The Netherlands; 4 Cancer Center Amsterdam, Emma Children’s Hospital, Amsterdam UMC, Vrije Universiteit, Amsterdam, The Netherlands

**Keywords:** Health-related quality of life, Computerized adaptive testing, Reliability, Validity, Psychometrics, Social functioning

## Abstract

**Purpose:**

This study aimed to validate the PROMIS Pediatric item bank v2.0 Peer Relationships and compare reliability of the full item bank to its short form, computerized adaptive test (CAT) and the social functioning (SF) subscale of the Pediatric Quality of Life Inventory (PedsQL™).

**Methods:**

Children aged 8–18 (*n* = 1327), representative of the Dutch population completed the Peer Relationships item bank. A graded response model (GRM) was fit to the data. Structural validity was assessed by checking item-fit statistics (S-*X*^2^, *p* < 0.001 = misfit). For construct validity, a moderately strong correlation (> 0.50) was expected between Peer Relationships and the PedsQL SF subscale. Cross-cultural DIF between U.S. and NL was assessed using logistic regression, where an item with McFadden’s pseudo *R*^2^ > 0.02 was considered to have DIF. Percentage of participants reliably measured was assessed using the standard error of measurement (SEM) < 0.32 as a criterion (reliability of 0.90). Relative efficiency ((1-SEM^2^)/*n*_items_) was calculated to compare how well the instruments performed relative to the amount of items administered.

**Results:**

In total, 527 (response rate: 39.7%) children completed the PROMIS v2.0 Peer Relationships item bank (*n*_*items*_ = 15) and the PedsQL™ (*n*_items_ = 23). Structural validity of the Peer Relationships item bank was sufficient, but one item displayed misfit in the GRM model (S-*X*^2^ < 0.001); 5152R1r (“I played alone and kept to myself”). The item 733R1r (“I was a good friend”) was the only item that displayed cross-cultural DIF (*R*^2^ = 0.0253). The item bank correlated moderately high (*r* = 0.61) with the PedsQL SF subscale Reliable measurements were obtained at the population mean and > 2SD in the clinically relevant direction. CAT outperformed all other measures in efficiency. Mean T-score of the Dutch general population was 46.9(SD 9.5).

**Conclusion:**

The pediatric PROMIS Peer Relationships item bank was successfully validated for use within the Dutch population and reference data are now available.

**Supplementary Information:**

The online version contains supplementary material available at 10.1007/s11136-021-02781-w.

## Introduction

Measuring patient-reported outcomes (PROs) has become increasingly important in healthcare for shared-decision making and value-based healthcare [1–4]. A more patient-centered approach to healthcare is possible by assessing self-reported daily functioning or symptoms of patients [5]. Patient-reported outcome measures (PROMs) are instruments used to measure PROs. However, PROMs measuring the same domains of functioning often vary in content, psychometric properties, and scoring methods. Due to these differences, domain scores are often incomparable between instruments and the interpretation of scores is unstandardized. Additionally, traditional domain scores apply classical test theory and are additive, whereas certain items should, based on their content, carry a stronger weight in calculating the domain score (e.g., “I have thought about ending my life” should have a stronger weight than “I felt sad” in a depressive symptoms questionnaire). To overcome these issues, the Patient-Reported Outcomes Measurement Information System (PROMIS®) initiative developed item banks for children and adults for generic, relevant domains of physical, social, and mental health [6–8]. Item banks are large selections of items that measure the same domain (e.g., relationships with peers) across a wide range of functioning. PROMIS item banks were developed using item-response theory modeling (IRT) [9]. IRT is a psychometric method where differences in item content can be taken into account when calculating sum scores, by applying item-specific difficulty and discrimination parameters. IRT provides the opportunity to scale items and persons onto a single metric, improving the interpretability of scores. By applying IRT modeling, the items are ordered by their difficulty and discriminative ability and this information is used to develop short forms and to apply computerized adaptive testing (CATs) [9]. With CAT, items are selected from an item bank (i.e., a large set of items that all measure the same construct) based on responses to previous items.

In pediatrics, CATs can improve the response rate of children when measuring patient outcomes in clinical practice or research. Previous research has shown that children have trouble with routinely completing PROMs due to the length and repetitive, irrelevant, or confrontational questions. CATs select questions that are more relevant to the level of functioning of the child and reduce the length of the questionnaire [7, 10, 11]. To implement pediatric PROMIS in the Netherlands, the pediatric Dutch-Flemish PROMIS group translated nine full PROMIS pediatric item banks (v1.0) [12] and validated them in a Dutch clinical sample of children with juvenile idiopathic arthritis [10]. Recently, additional PROMIS pediatric item banks/scales were developed in the U.S. (Sleep-Related Impairment, Sleep Disturbance [13] & Global Health [14]) and several item banks were updated to version 2.0 with new items and scoring methods. The pediatric Dutch-Flemish PROMIS group translated the new items for the v2.0 item banks in 2017 using the standard PROMIS translation procedure (see Haverman et al. [12] for a detailed description of the translation procedure). However, before the PROMIS pediatric v2.0 item banks can be implemented as CATs, the validity and reliability of the updated items banks have to be investigated.

The current study is part of a larger cross-sectional study that aims to investigate the psychometric properties of multiple PROMIS pediatric v2.0 item banks in a representative sample of the Dutch general population and to obtain reference data. This paper presents a description of the data collection procedure and the validation of the PROMIS pediatric v2.0 Peer Relationships item bank.

## Methods

### Procedure and participants

Data were collected of children (8–12 years old) and adolescents (13–18 years old) between December 2017 and April 2018 by marketing agency Kantar Public. The goal of the data collection was to obtain representative data of approximately 550 participants for nine PROMIS pediatric item banks. A two-step random stratified sampling method was used to ensure that the child and adolescent samples were representative (within 2.5% of the Dutch population) on key demographics; sex, age, ethnicity, social class, and educational level (the latter only for adolescents). The first step was to randomly draw participants from each demographic stratum (representing a subpopulation), with an expected response rate of 50% for all strata. Subsequently, actual response rates were calculated and used to adjust the amount of participants drawn from the same strata in the second step. To limit the burden of completing questionnaires, two item bank batteries (A and B) were assembled with equal administration times. Battery A contained the PROMIS pediatric Fatigue, Peer Relationships, Anger, Sleep-Related Impairment, Sleep Disturbance, and Sleep Practices item banks. Battery B contained the Pain Interference, Mobility, and Upper Extremity item banks. Both batteries contained a general sociodemographic questionnaire (parent-reported), the Pediatric Quality of Life Inventory (PedsQL 4.0), and PROMIS Global Health (v1.0, 7 + 2) scale. Participants were randomly assigned to one of the two batteries. Partial completion of a test battery was not possible, as online administration through the panel did not log results until the entire test battery was administered.

E-mails were sent to the parents of 2654 children with a login code that granted access to the research website (onderzoek.hetklikt.nu/promis). Informed consent was provided by parents (children aged 8–15) and adolescents (aged ≥ 12 years). The data collection was approved by the Medical Ethics Committee of the Amsterdam UMC, location AMC.

In total, a representative sample of 1098 children completed the item bank battery they were assigned to (response rate of 41.37%). The sociodemographic characteristics of the final samples were provided by Kantar and were subsequently compared to the general population, which can be seen in Online Appendix A.

### Measures

#### Sociodemographic questionnaire

Parents completed a sociodemographic questionnaire about themselves (age, country of birth, and educational level) and their child (age, gender, educational level (only for adolescents) and the presence of any chronic health conditions). For parents, the educational level was divided into low (primary, lower vocational, lower general, and middle general education), middle (middle vocational, higher secondary, and pre-university education), and high (higher vocational education, university).

#### PROMIS pediatric Peer Relationships item bank

The PROMIS pediatric v2.0 item bank Peer Relationships [15] is a 15-item item bank for children aged 8–18 assessing aspects of social participation and the quality of relationships with friends and acquaintances. Participants respond to items (e.g., “I spend time with my friends”) over the past 7 days. Item responses range from 1 (“Never”) to 5 (“Always”). The standard Peer Relationships static short form 8a contains eight items. The responses to these items were extracted from the completed full item bank. Domain scores for the full item bank and short form were calculated by applying the item parameters from the U.S. IRT model to the responses and calculating an estimate for the level of peer relationships (theta; θ). This estimate was transformed into a *T*-score where 50 is the mean of the U.S. general population with a standard deviation of 10. A higher score represents better relationships with peers.

#### Pediatric quality of life inventory (4.0)

The PedsQL 4.0 is a generic 23-item questionnaire that assesses the self-reported Health-Related Quality Of Life (HRQOL) of children (aged 8–18 years) [16]. It contains items retaining to four domains of HRQOL; physical health (8 items), emotional functioning (5 items), social functioning (5 items), and school functioning (5 items). The PedsQL utilizes a recall period of one week and the items (e.g., “Other kids/teens do not want to be my friend”) are scored from 1 (“Never a problem”) to 5 (”Almost always a problem”). The response options are transformed into values of 0, 25, 50, 75, and 100, where a higher score represents better functioning on the item. Domain scores are calculated as the mean of all items in a specific domain (range 0–100, higher score represents better functioning). The total PedsQL score is calculated by the mean of all items of the entire questionnaire (range 0–100). The PedsQL has been validated for use in clinical practice in the Netherlands [17].

### Statistical analyses

#### Structural validity

To assess the structural validity of the PROMIS Peer Relationships item bank, a graded response model (GRM) was fitted. A GRM is an IRT model for items with ordinal response categories and requires several assumptions to be met: unidimensionality, local independence, and monotonicity. A confirmatory factor analysis (CFA) with weighted least square mean- and variance-adjusted (WLSMV) estimator was performed to assess *unidimensionality* using the R-package “lavaan (v0.6–3)” [18]. We used the following criteria for an acceptable CFA fit: Scaled Comparative Fit Index (CFI) and Tucker–Lewis Index (TLI) values > 0.95, a standardized root mean square residual (SRMR) value < 0.10, and a root mean square error of approximation (RMSEA) value < 0.08 [19]. If CFA fit did not meet these criteria, a bi-factor model was fit to assess if unidimensionality was sufficient to continue IRT analyses, by assessing if the hierarchical omega (*ω*_h_) was > 0.80 and the explained common variance (ECV) > 0.60. *Local independence* was assessed by looking at the residual correlations in the CFA model. An item pair was considered to be locally independent if the residual correlation was < 0.20 [20]. Finally, *monotonicity* was assessed using Mokken scaling [21, 22]. The assumption of monotonicity was considered met when the item H values of all items were ≥ 0.30 and the H value of the entire scale was ≥ 0.50.

Once the assumptions were met, a GRM was fitted to estimate item discrimination and threshold (difficulty) parameters, using the Expectation–Maximization (EM) algorithm within the R-package “mirt (v1.29)” [23]. The *discrimination parameter* (*α*) represents the ability of an item to distinguish between patients with a different level of relationships with peers (*θ*). The *threshold parameters* (*β*) represent the required level of peer relationships of a person to choose a higher response category over a lower response category, hence there is always one less threshold than the amount of response categories for each item. To assess item fit, the differences between observed and expected responses under the GRM were calculated using the S-X^2^ statistic [24]. A *p* value of the S-*X*^2^ statistic < 0.001 for an item is considered as item misfit [20]. When item misfit was present, item-fit plots were assessed. Item-fit plots rank participants from lowest to highest levels of functioning, divide the participants into ten blocks, and then average the responses on one item per block. This results in a smooth line graph, while accounting for a reasonable bias/variance trade-off [23]. If the item fits well, higher theta scores should lead to higher responses on the item (on average).

#### Construct validity

To assess construct validity, the Peer Relationships T-score was correlated with the four PedsQL subscales scores. A moderately high correlation (Pearson’s *r* > 0.50) was expected between the PROMIS Peer Relationships T-score and the PedsQL social functioning subscale score [10, 25, 26]. Lower correlations (Δ*r* > 0.10) were expected with the three other PedsQL subscale scores (emotional, physical, and school functioning). Construct validity was considered sufficient if 75% of the hypotheses were met.

#### Cross-cultural validity

For assessing cross-cultural validity, our sample was compared to the U.S. calibration sample (*n* = 5689) that was used for estimating the U.S. item parameters [15], obtained from the HealthMeasures Dataverse [27]. The U.S. calibration sample contained 5689 participants (1463–2518 responses on each item) and consisted of a combination of chronically ill children (22.7%) and children from the general population. To evaluate differences in item parameters between the Dutch and U.S. samples, differential item functioning (DIF) was assessed with the R-package “lordif (v0.3–3)” [28]. Two types of DIF were considered: uniform, when the DIF is consistent across the scale (i.e., the item thresholds differ between the groups), and non-uniform DIF, when DIF varies across the scale (i.e., discrimination parameters differ between the groups) [29]. DIF was evaluated between the Dutch and the U.S. calibration sample, with the McFadden’s pseudo *R*^2^, where a *R*^2^ ≥ 0.02 indicated DIF.

#### Reliability

In IRT, each response pattern results in a different level of functioning (*θ*) and an associated reliability, expressed as the standard error of theta (SE(*θ*)). A SE(*θ*) of 0.32 or lower was considered a reliable measurement, which corresponds to a reliability of 0.90 or higher. To investigate the reliability of the Peer Relationships item bank and short form, *θ* estimates and SE(*θ*) were calculated using the Expected A Posteriori (EAP) estimator. Post hoc CAT simulations were performed on the respondent data with the R-package “catR (v3.16)” [30] using maximum posterior weighted information (MPWI) selection criterion and EAP estimator [31] to assess how a CAT would perform when applying the Dutch model parameters. The starting item was the item that offered most information at the mean of the study sample (*θ* = 0). The stopping rules for the CAT were a maximum of eight items administered (which is equal to the length of the short form) or a SE(*θ*) < 0.32 [32]. To compare the reliability of the full item bank, short form, and CAT with the PedsQL social functioning scale, a GRM model was also fit to the PedsQL data and *θ* estimates and SE(*θ*) were calculated and presented in a reliability plot. In a reliability plot, each line represents the standard errors of measurement across *θ* or T-score of one measure. A lower line is indicative of a higher reliability. Plotted dots are individual estimated thetas or T-scores and their associated standard errors of measurement resulting from post hoc CAT simulations. The current PROMIS convention is to use the U.S. parameters model for calculating *T*-scores, unless significant differences are found between country-specific model parameters and the U.S. parameters. Therefore, the reliability of measurements were also calculated using the U.S. parameters (provided by HealthMeasures) and plotted in a reliability plot and included the T*-*score distribution of the Dutch population as histogram. In addition, efficiency of measures was calculated for each participant by dividing the total test information by the amount of items administered. To compare PROMIS measures (full item bank, short form, and CAT), the relative efficiency between measures was calculated by dividing the mean efficiency of one measure by the other. The mean (SD) T-score of the Dutch population was calculated based on the U.S. parameters. Using percentiles good (≥ 26th percentile), fair (6–25th percentiles), and poor (≤ 5th percentile) functioning cut-offs were determined, in accordance with recently defined U.S. cut-offs for this item bank (personal communication C. Forrest, data submitted).

## Results

Based on parent reports, several respondents (*n* = 16) were removed as they were either too young (< 8) or too old (> 18) to be included in this study. In total 527 (response rate of 39.7%), participants completed the battery that included the Peer Relationships item bank and 483 participants (only children aged 8 to 17) completed the PedsQL 4.0. Their sociodemographic characteristics are presented in Table 1. There was no missing data.

**Table 1:** Sociodemographics of the Peer Relationships item bank sample for the main analyses and the relative efficiency analysis

Sociodemographics	Main analysis sample (*n* = 527)	Relative efficiency analysis^a^ (*n* = 483)
Age (years)	13.59 (3.08)	13.14 (2.81)
Gender		
Female	255	235
Male	272	248
Ethnicity		
Dutch	436	402
Non-western immigrants	27	23
Western immigrants	64	58
Educational level (parent)		
Low	72	64
Middle	255	229
High	200	190

^a^Used for calculating relative efficiency between the PROMIS item bank, short form, CAT, and the PedsQL social functioning subscale. 18-year olds did not complete the PedsQL

### Structural validity

**Fig. 1:**
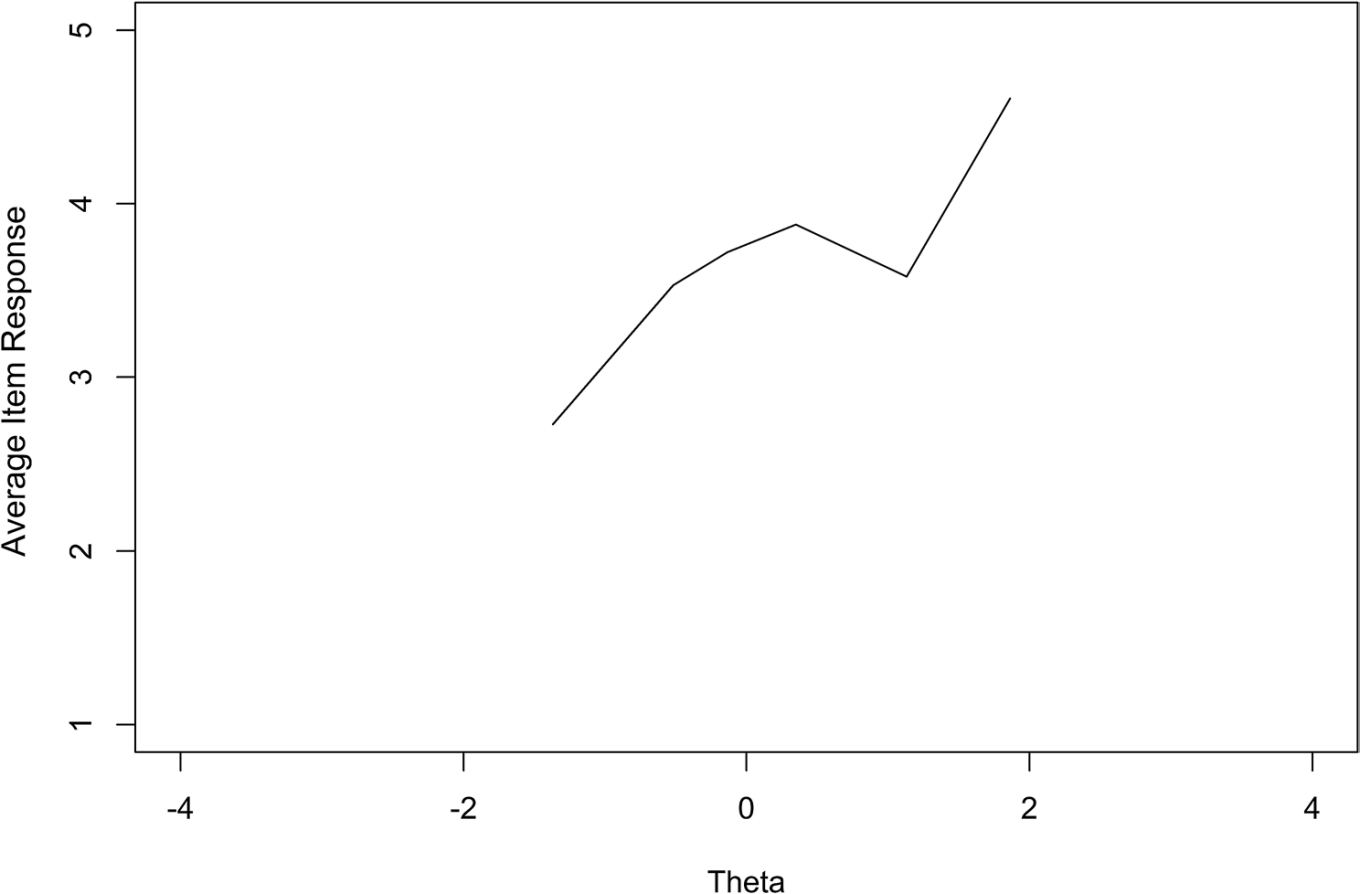
Average item response across the range of theta for the item “I played alone and kept to myself”

The data satisfied all assumptions for fitting a GRM. Unidimensionality (see Online Appendix B) was initially not satisfied by the CFA (CFI = 0.95, TLI = 0.94, RMSEA = 0.14, SRMR = 0.06), but the bi-factor model indicated that the data were unidimensional enough for subsequent IRT analyses (*ω*_h_ = 0.87, ECV = 0.80). There were no items with local independence and the entire item bank displayed sufficient monotonicity (*H*_i_ > 0.30, *H* > 0.60). One item displayed item misfit, this was the item “I played alone and kept to myself” (S-*X*^2^ < 0.001). The item-fit plot, which displays the average response of participants across their theta estimates, is shown in Fig. 1.

### Construct validity

The *T-*score of the Peer Relationships item bank had a moderately high correlation (*r* = 0.61) with the PedsQL social functioning subscale sum score. Correlations with the physical, emotional, and school functioning subscales were 0.30, 0.41, and 0.38, respectively. All hypotheses regarding construct validity were met.

### Cross-cultural validity

One item, 733R1r (“I was a good friend”), displayed uniform DIF (*R*^*2*^ = 0.0253) between the Dutch and U.S. samples. Dutch participants score lower on this item compared to U.S. participants with the same levels of functioning.

### Reliability

**Fig. 2:**
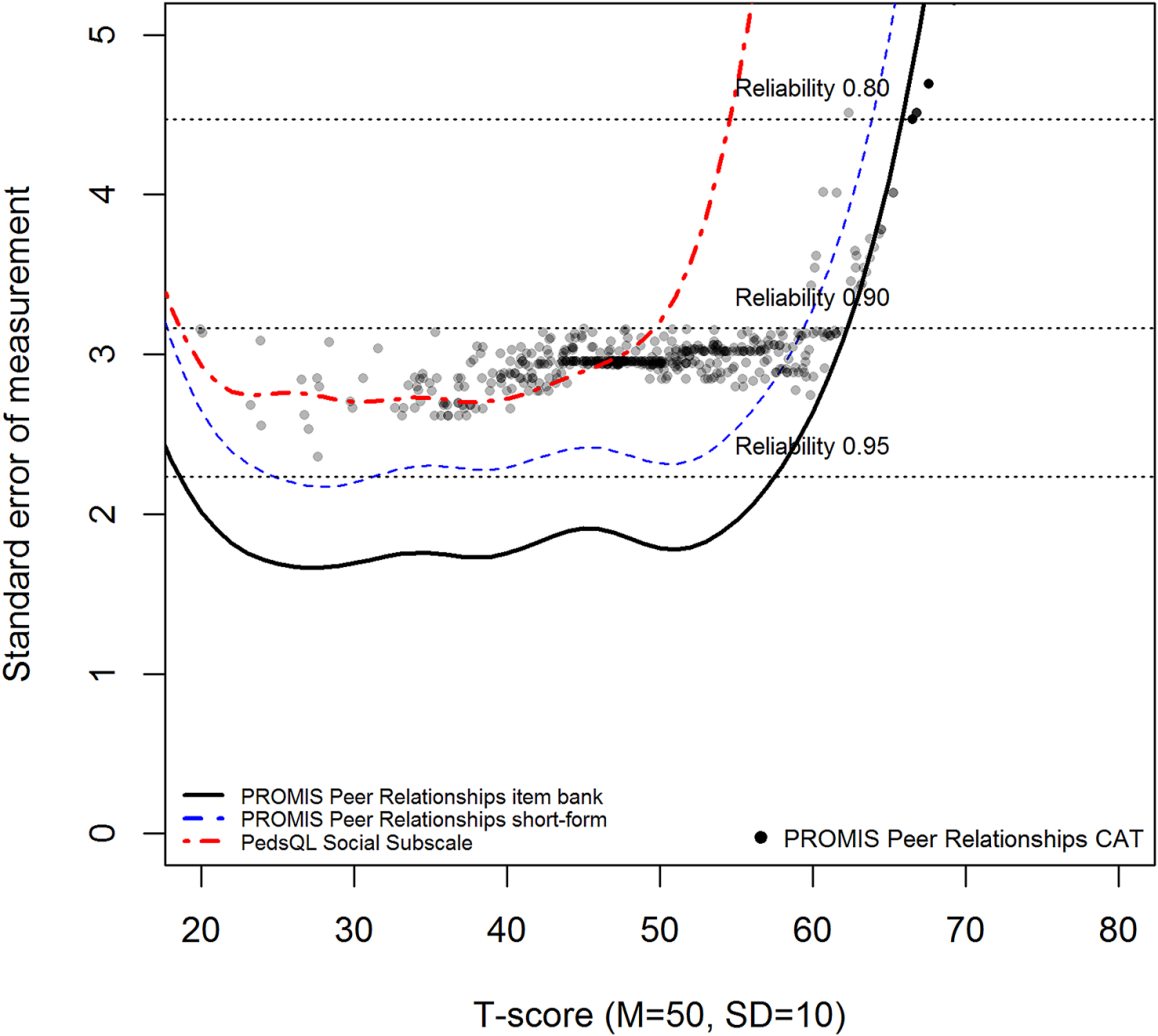
Standard error of measurement (SE(*θ*)) of the full item bank, short form, and CAT of the PROMIS Peer Relationships item bank and the PedsQL social functioning subscale, using the Dutch model parameters

The model based on the Dutch parameters (see Online Appendix C; range *a* = 0.7–3.7, range *B*_*1-min*_* – B*_*4-max*_ =  − 3.8 to 2.0) provided reliable measurements at the mean of the sample (*θ* = 0) and more than two standard deviations in the clinically relevant direction. Compared to the PedsQL social functioning subscale, all PROMIS Peer Relationships measures were more reliable (see Fig. 2). The majority of respondents were reliably estimated by the full item bank (87.7%), short form (81.6%), and post hoc CATs (82.7%; see Table 2). The measurement efficiency of the CAT outperformed the PROMIS full item bank, short form, and the PedsQL social functioning subscale (see Table 3).

**Table 2:** Reliability of measurements for the full item bank (FL), short forms (SF), and computerized adaptive test (CAT) of the PROMIS pediatric Peer Relationships item bank in the general Dutch population (*n* = 527)

Item Bank	Mean FL SE(θ)	FL SE(*θ*) < 0.32* %	Mean SF SE(θ)	SF SE(*θ*) < 0.32* %	Mean CAT SE(θ)	CAT SE(*θ*) < 0.32* %	Mean CAT items administered	FL amount of items	SF amount of items
PROMIS Peer Relationships (DF)	0.227	87.7	0.290	81.6	0.322	82.7	5.1	15	8
PROMIS Peer Relationships (US)	0.300	75.1	0.360	49.3	0.362	51.4	7.4	15	8

*SE(θ)* standard error of measurement, *FL* full item bank, *SF* short form, *CAT* computerized adaptive testing, *DF* Dutch-Flemish, *US* United States

*Percentage of participants that were measured reliably (< 0.32 SE(*θ*))

**Table 3:** Relative efficiency of the PROMIS Peer Relationships full item bank, short form, CAT compared to the social functioning subscale of the PedsQL (*n* = 527)

	PROMIS Peer Relationships full item bank	Peer Relationships short form	Peer Relationships CAT
PedsQL social functioning	.97*	.87*	.69*
Peer Relationships full item bank	–	.90	.70
Peer Relationships short form	–	–	.78

*Based on *n* = 483; A relative efficiency ratio < 1 indicates that the row has a lower efficiency than the column

**Fig. 3:**
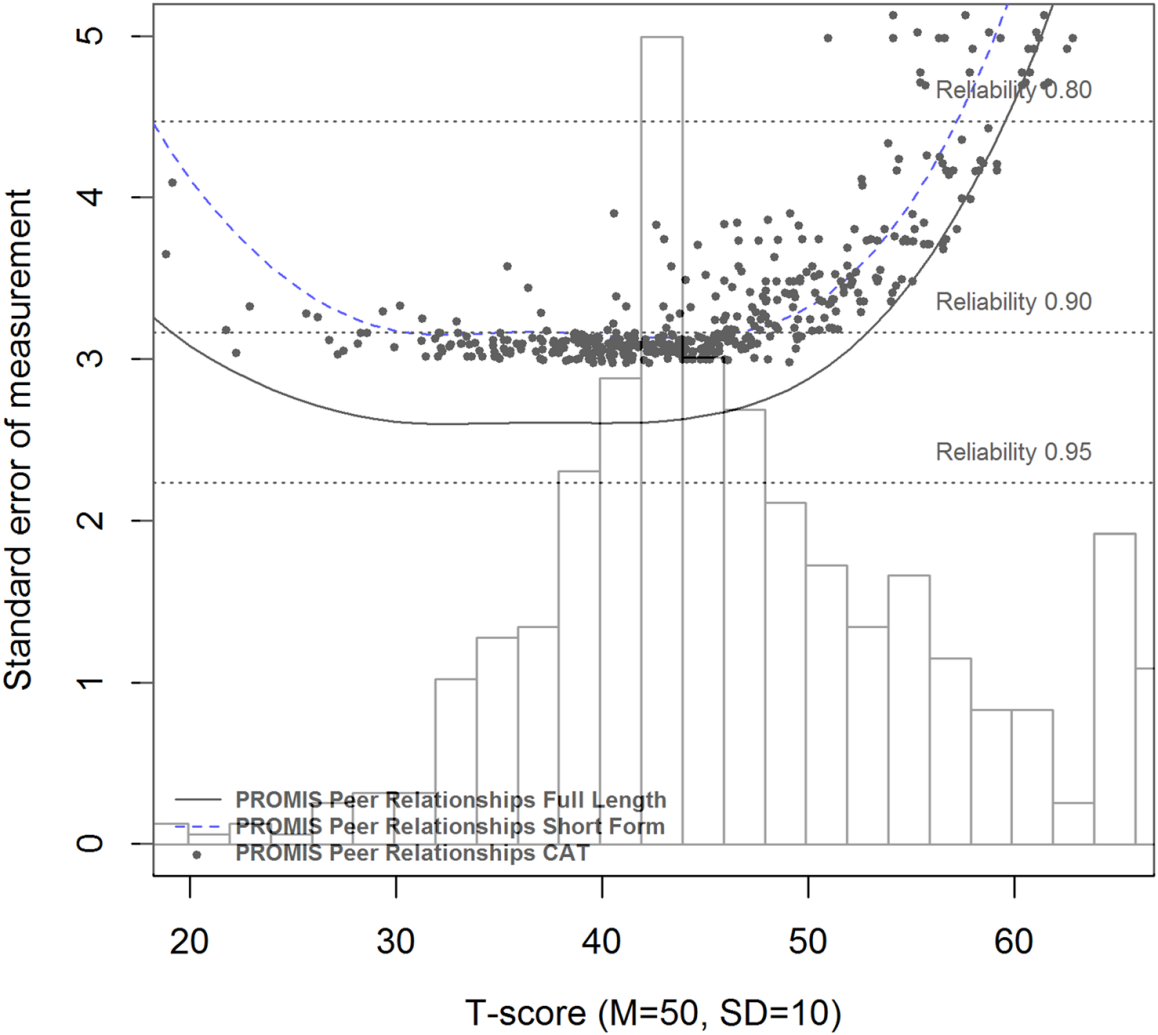
Standard error of measurement (SE(*θ*)) of the full item bank, short form, and CAT of the PROMIS Peer Relationships item bank, using the U.S. model parameters and the distribution of the Dutch sample *T*-scores plotted as histogram

With the U.S. parameters, reliable scores were obtained at the sample mean and in more than two standard deviations in the clinically relevant direction, however, fewer participants were measured reliably than with the Dutch parameters for the full item bank (75.1% vs. 87.7%), short form (41.9% vs. 81.6%), and post hoc CATs (51.4% vs. 82.7%). More CAT items were required when applying the US parameters (mean number of items = 7.4) than when using the Dutch parameters (mean number of items = 5.1). The distribution of Dutch *T*-scores, based on the U.S. parameters, and the reliability of the full item bank, short form, and post hoc CATs based on the U.S. model are shown in Fig. 3. The mean *T*-score of the Dutch sample was 46.9 (SD 9.5). A T-score ≥ 41.1 indicates good functioning, T-scores between 33.4 and 41.0 indicate fair functioning and ≤ 33.3 is indicative of poor functioning.

## Discussion

This is the first study that assessed the psychometric properties of a PROMIS pediatric item bank in a representative general population sample outside of the U.S.. The Peer Relationships item bank performed sufficiently in the Dutch general population. Structural validity was sufficient as all but one item (5152R1r; “I played alone and kept to myself”) fit the IRT model well. One item (733R1r; “I was a good friend”) displayed cross-cultural DIF. Construct validity was also sufficient, as the item bank correlated moderately high with the PedsQL social functioning subscale. The item bank measures reliably at the mean of the Dutch population and more than two standard deviations in the clinically relevant direction. This study also displayed that CAT administration of PROMIS item banks outperforms the full item bank and short form in terms of efficiency.

The results found in this study were similar to the results of the original development study of the Peer Relationships item bank in the U.S. [15]. Similar values were found for unidimensionality and item fit. Model parameters were similar, although higher discrimination parameters were found in the Dutch model. There was a single exception, the item 5152R1r (“I played alone and kept to myself”) did not perform well in the Dutch model. It displayed poor item fit and a low discriminative ability (*a* = 0.78). Analyzing the currently available U.S. data [27] resulted in misfit for this item as well. The item plot displayed that mainly participants with high theta values had a low mean response to this specific item. This is possibly due to this item being the only item in the item bank that is negatively phrased, thus participants who continuously marked the response category furthest to the right may have accidently selected the lowest response option on this item as item scores were reversed. In the study of DeWalt et al. [15], where the misfit was not reported, response categories (i.e., “Never” to “Almost Always”) were repeated in the header on the second page, just before the item with misfit. This was not the case in the current study. We recommend users of this item bank to pay attention to the lay-out of this item in future applications. The item 733R1r (“I was a good friend”) displayed cross-cultural DIF. It is possible that the concept of a “good friend” is different between cultures. Therefore, it may be adequate to use country-specific item parameters for this item.

An interesting finding is that the Dutch IRT model provided more reliable measurements and required fewer items with CATs than the U.S. model. The Dutch discrimination parameters were generally higher than the discrimination parameters of the U.S. model. Higher discrimination parameters result in more reliable measurements. Differences were found in the distribution of T-scores in the Dutch versus U.S. population, which may explain these differences in parameters. Although DIF was not found with the “lordif” package in R, we suspected that with the differences found in discrimination parameters there may have been more DIF than we initially discovered. Therefore, we ran additional DIF analyses using “IRTPRO” [33], which uses a two-step Wald approach for detecting DIF, instead of the logistic ordinal regression approach performed by “lordif.” This resulted in every item in the item bank displaying DIF (see Online Appendix D), however, previous simulation studies have indicated Type 1 errors while using two-step Wald approach for detecting DIF [34]. Subsequently, we anchored the three items with the lowest DIF to put the remaining items onto the same scale (partial purification [35]), but the differences in the discrimination parameters persisted. Possible causes of these differences could be the mode of administration (in-person versus online), differences in representativeness of the sample, or the inclusion of patients with chronic illnesses, which was only done in the U.S. sample. Our conclusion is that, regardless of DIF, the differences in discrimination parameters resulted in more participants being reliably measured when using the set of parameters with higher discriminatory parameter values (in this case the Dutch parameters). As this could have further implications for model selection (U.S. or Dutch parameters) when administering CATs, it is advisable to investigate the differences of the two IRT models within a more comparable sample, for example, a bilingual sample. If item parameter differences persist in these comparisons, selecting the parameters with highest discriminatory parameters would be advised in the Netherlands, as to provide more reliable measurements in fewer items administered by CAT.

This study contained several limitations. Due to the sample being representative of the Dutch general population, it contained mainly healthy participants. This lead to a subgroup of participants (6.3%) that responded “Almost Always” to all items in the item bank. While this has no substantial effect on item parameter estimates [36], as the subgroup is quite small, these participants could not be measured reliably as they had no variance in responses. This finding could indicate that the item bank requires more difficult items at the high-end of the scale to reliably measure these participants.

Another limitation is that the PROMIS Peer Relationships item bank and the PedsQL social functioning subscale do not entirely measure the same construct [33], which is preferable for assessing construct validity. Our finding of a moderately high correlation is consistent with the findings of DeWalt et al. [15], who could not develop a unidimensional model without separating relationships with peers from social functioning. The PedsQL social functioning subscale contains relatively more items about keeping up with other children/adolescents and being shut out from activities with others, whereas the Peer Relationships item bank focuses more on the quality of relationships with peers. No other legacy instrument was found that accurately represented the same domain as assessed by the Peer Relationships item bank, thus the PedsQL social functioning subscale was considered most suitable for evaluating construct validity.

The aim of the Dutch-Flemish PROMIS group is to implement PROMIS (CATs) into research and clinical practice, by translating and validating item banks and providing reference data for comparison. After previously validating the pediatric item banks in a clinical population [10], this study provides evidence that the PROMIS pediatric v2.0 item bank Peer Relationships performs sufficiently in the general Dutch population and can now be used as full item bank, short form, or CAT in the Netherlands through the Dutch-Flemish Assessment Center (www.dutchflemishpromis.nl).

## Supplementary Information

Below is the link to the electronic supplementary material.

Supplementary file1 (PDF 740 KB)

## Data Availability

Data may be made available upon a reasonable request.

## Code availability

Custom code will not be made available.
