# Supplementary material for: Psychometric properties of the Patient-Reported Outcomes Measurement Information System (PROMIS®) pediatric item bank peer relationships in the Dutch general population
Source: Qual Life Res. 2021 Feb 19;30(7):2061–70. doi: 10.1007/s11136-021-02781-w (PMC8233291; doi:10.1007/s11136-021-02781-w)
Supplement: Supplementary file 1 — Supplementary file1 (PDF 740 KB) [file 11136_2021_2781_MOESM1_ESM.pdf]

Supplementary Material

Belonging to: Psychometric properties of the Patient-Reported Outcomes Measurement Information System (PROMIS®) Pediatric item bank Peer Relationships in the Dutch general population (Luijten et al., 2021).

Contents

Appendix A – Sociodemographic characteristics of the pediatric and adolescent study samples in comparison to the general population.

Appendix B – Assessment of unidimensionality through confirmatory factor analysis and bi-factor analyses.

Appendix C – Estimated item parameters and item fit of the pediatric PROMIS Peer Relationships item bank in the Dutch general population (n=527).

Appendix D – Raw output of IRTPRO DIF analyses.

Appendix A – Sociodemographic Characteristics

Appendix A. Sociodemographic characteristics of the pediatric and adolescent study samples in comparison to the general population.

	8 – 12 years				13 – 18 years		
**Pediatric study sample (N=492)**	**Sample (*N*)**	**Sample (%)**	**Dutch Population* (%)**	**Adolescent study sample (N=606)**	**Sample (*N*)**	**Sample (%)**	**Dutch Population* (%)**
**Age (years)**				**Age (years)**			
8	90	18.3	19.1	13	101	16.7	16.9
9	92	18.7	19.4	14	106	17.5	17.2
10	98	19.9	20.0	15	106	17.5	16.8
11	104	21.1	20.7	16	100	16.5	16.7
12	108	22.0	20.8	17	86	14.2	16.2
				18	107	17.7	16.3
**Gender**							
Male	241	51.0	51.1		316	52.1	51.1
Female	251	49.0	48.9		290	47.9	48.9
**Ethnicity**							
Dutch	386	78.5	76.0		510	84.2	76.3
Non-western immigrants	85	17.3	17.4		63	10.4	16.9
Western immigrants	21	4.3	6.6		33	5.4	6.8
				**Educational Level**			
				Elementary[1]	6	1.0	0.6
				Lower vocational LBO/VMBO[1]	149	24.6	23.2
				Lower vocational MAVO[1]	189	31.2	30.1
				Secondary vocational MBO[2]	118	19.5	20.1
				General secondary education HAVO/VWO[3]	129	21.3	21.8
				HBO/WO Bachelor[3]	15	2.5	4.1
				WO Master or Doctorate[3]	0	0.0	0.2

Note: * = Based on the Gold Standard 2017 (Statistics Netherlands; www.cbs.nl/en-gb) population numbers; [1], Low educational level; [2], Intermediate educational level; [3], High educational level.

Appendix B – Unidimensionality Analyses

Appendix B. Assessment of unidimensionality through confirmatory factor analysis (CFA) and bi-factor analyses.

	Factor loadings				
**Items**	**Unidimensional CFA**	**Bi-factor G**	**Bi-factor F1**	**Bi-factor F2**	**Bi-factor F3**
I felt accepted by other kids my age.	0.82	0.72	0.09	0.16	0.17
I was able to count on my friends.	0.88	0.77	-0.01	-0.01	0.55
I was able to talk about everything with my friends.	0.83	0.73	0.04	0.06	0.35
I was good at making friends.	0.81	0.73	0.12	0.19	0.07
My friends and I helped each other out.	0.89	0.80	0.10	0.05	0.28
Other kids wanted to be my friend.	0.85	0.68	-0.01	0.54	0.03
Other kids wanted to be with me.	0.89	0.70	-0.01	0.59	0.00
Other kids wanted to talk to me.	0.87	0.71	0.04	0.47	0.00
I felt good about my friendships.	0.88	0.80	0.15	0.08	0.14
I liked being around other kids my age.	0.80	0.73	0.18	0.11	0.01
I played alone and kept to myself.	0.39	0.38	0.14	-0.04	-0.01
I shared with other kids (food, games, pens, etc.).	0.71	0.62	0.13	0.21	-0.04
I spent time with my friends.	0.84	0.79	0.21	0.07	0.02
I was a good friend.	0.81	0.74	0.23	-0.01	0.00
I was able to have fun with my friends.	0.88	0.83	0.26	-0.05	0.02
	**Fit Indices**				
**CFA**					
Scaled CFI	0.95				
Scaled TLI	0.94				
Scaled RMSEA	0.15				
**Bi-factor**					
Omega H	0.88				
ECV	0.81				

Note: G; General factor; F1-F3, random factors.

Appendix C – Item Parameters and Item Fit Statistics

Appendix C. Estimated item parameters and item fit of the pediatric PROMIS Peer Relationships item bank in the Dutch general population (*n*=527).

Item	Item Parameters and item fit statistics								DIF
	α	β_1	β_2	β_3	β_4	S-X^2	Df	P	R^2
**PROMIS Peer Relationships Item Bank**									
I felt accepted by other kids my age.	2.82	-2.79	-2.20	-1.22	0.04	53.52	37	0.04	0.011
I was able to count on my friends.	3.18	-2.56	-2.07	-1.11	0.09	43.31	36	0.19	0.011
I was able to talk about everything with my friends.	2.58	-2.69	-1.81	-0.73	0.31	40.68	43	0.57	0.014
I was good at making friends.	2.67	-2.22	-1.56	-0.67	0.33	34.66	47	0.91	0.000
My friends and I helped each other out.	3.72	-2.82	-2.19	-1.10	0.12	33.64	31	0.34	0.003
Other kids wanted to be my friend.	2.50	-2.41	-1.54	-0.27	0.91	59.54	47	0.10	0.001
Other kids wanted to be with me.	2.76	-2.32	-1.60	-0.40	0.96	50.83	42	0.16	0.004
Other kids wanted to talk to me.	2.83	-2.97	-1.90	-0.68	0.63	33.67	38	0.67	0.001
I felt good about my friendships.	3.57	-2.56	-1.83	-1.12	0.12	39.59	34	0.23	0.000
I liked being around other kids my age.	2.56	-2.60	-2.01	-0.94	0.37	51.08	40	0.11	0.005
I played alone and kept to myself.	0.78	-3.76	-2.37	-0.54	2.00	140.52	82	0.00*	0.006
I shared with other kids (food, games, pens, etc.).	1.96	-2.78	-1.83	-0.66	0.79	77.36	53	0.02	0.000
I spent time with my friends.	3.02	-2.37	-1.73	-0.75	0.58	34.97	38	0.61	0.000
I was a good friend.	2.73	-3.23	-2.35	-1.20	0.28	48.02	35	0.07	0.025^
I was able to have fun with my friends.	3.69	-2.81	-2.27	-1.21	0.02	27.25	27	0.45	0.004

Note: DIF; Differential item functioning between Dutch and U.S. parameters as measured by McFadden's Pseudo R^2 Rα; discrimination parameter, β_{1-4}; threshold parameters, *; Significant at p < 0.001, ^; Weak effect

Appendix D – Raw output of IRTPRO DIF Analyses

IRTPRO Version 4.2
Output generated by IRTPRO estimation engine Version 5.20 (64-bit)

Project:	
Description:	
Date:	08 June 2020
Time:	04:35 PM

Table of Contents

Graded Model Item Parameter Estimates for Group 1, logit: $a(\theta - b)$

Summed-Score Based Item Diagnostic Tables and X^2s for Group 1

Graded Model Item Parameter Estimates for Group 2, logit: $a(\theta - b)$

Summed-Score Based Item Diagnostic Tables and X^2s for Group 2

Group Parameter Estimates

DIF Statistics for Graded Items

Marginal fit (X^2) and Standardized LD X^2 Statistics for Group 1

Marginal fit (X^2) and Standardized LD X^2 Statistics for Group 2

Item Information Function Values for Group 1 at 15 Values of θ from -2.8 to 2.8

Item Information Function Values for Group 2 at 15 Values of θ from -2.8 to 2.8

Likelihood-based Values and Goodness of Fit Statistics

Summary of the Data and Control Parameters

Graded Model Item Parameter Estimates, logit: $a\theta + c$

Item	Label		a	s.e.		c_1	s.e.		c_2	s.e.		c_3	s.e.		c_4	s.e.
1	P5018R1r_PEER	5	2.12	0.10	1	4.36	0.14	2	3.61	0.12	3	1.97	0.08	4	0.42	0.06
2	P5058R1r_PEER	10	1.92	0.09	6	4.07	0.13	7	3.55	0.11	8	1.61	0.07	9	0.11	0.06
3	P5056R1r_PEER	15	1.71	0.07	11	3.54	0.11	12	2.72	0.09	13	0.76	0.06	14	-0.50	0.06
4	P1147R1r_PEER	20	2.08	0.10	16	4.97	0.17	17	4.14	0.14	18	2.05	0.08	19	0.42	0.06
5	P5055R1r_PEER	25	2.12	0.10	21	5.17	0.19	22	4.47	0.16	23	2.24	0.09	24	0.60	0.06
6	P233R2r_PEER	30	2.04	0.10	26	4.47	0.15	27	3.57	0.13	28	1.37	0.07	29	-0.59	0.06
7	P210R1r_PEER	35	2.37	0.11	31	5.36	0.19	32	4.31	0.15	33	1.58	0.08	34	-0.51	0.07
8	P9020R1r_PEER	40	2.23	0.11	36	5.47	0.19	37	4.32	0.15	38	1.98	0.08	39	-0.04	0.06
9	P726aR2r_PEER	45	1.83	0.12	41	4.98	0.22	42	4.41	0.19	43	2.62	0.12	44	1.12	0.08
10	P9019r_PEER	50	1.89	0.12	46	4.92	0.22	47	4.30	0.18	48	2.23	0.11	49	0.86	0.08
11	P5152R1r_PEER	55	0.82	0.07	51	3.16	0.12	52	2.25	0.09	53	0.73	0.06	54	-0.32	0.06
12	P5150R1r_PEER	60	1.28	0.08	56	3.44	0.13	57	2.84	0.10	58	1.09	0.07	59	-0.26	0.06
13	P5052R1r_PEER	65	1.60	0.10	61	4.19	0.17	62	3.48	0.14	63	1.65	0.08	64	-0.09	0.07
14	P733R1r_PEER	70	1.71	0.11	66	5.53	0.25	67	5.20	0.23	68	3.21	0.13	69	1.43	0.09
15	P2964R1r_PEER	75	2.27	0.16	71	5.39	0.25	72	4.80	0.22	73	3.18	0.15	74	1.52	0.10

Graded Model Item Parameter Estimates for Group 1, logit: $a(\theta - b)$ (Back to TOC)

Item	Label		a	s.e.	b_1	s.e.	b_2	s.e.	b_3	s.e.	b_4	s.e.
1	P5018R1r_PEER	5	2.12	0.10	-2.06	0.07	-1.71	0.06	-0.93	0.04	-0.20	0.03
2	P5058R1r_PEER	10	1.92	0.09	-2.12	0.08	-1.85	0.07	-0.84	0.04	-0.06	0.03
3	P5056R1r_PEER	15	1.71	0.07	-2.07	0.08	-1.59	0.06	-0.45	0.03	0.29	0.03
4	P1147R1r_PEER	20	2.08	0.10	-2.39	0.09	-1.99	0.07	-0.98	0.04	-0.20	0.03
5	P5055R1r_PEER	25	2.12	0.10	-2.44	0.09	-2.11	0.08	-1.06	0.04	-0.28	0.03
6	P233R2r_PEER	30	2.04	0.10	-2.19	0.08	-1.75	0.06	-0.67	0.03	0.29	0.03
7	P210R1r_PEER	35	2.37	0.11	-2.26	0.08	-1.82	0.06	-0.67	0.03	0.22	0.03
8	P9020R1r_PEER	40	2.23	0.11	-2.45	0.09	-1.94	0.07	-0.89	0.04	0.02	0.03
9	P726aR2r_PEER	45	1.83	0.12	-2.72	0.14	-2.40	0.12	-1.43	0.07	-0.61	0.04
10	P9019r_PEER	50	1.89	0.12	-2.61	0.14	-2.28	0.11	-1.18	0.06	-0.46	0.04
11	P5152R1r_PEER	55	0.82	0.07	-3.87	0.31	-2.76	0.22	-0.89	0.09	0.39	0.07
12	P5150R1r_PEER	60	1.28	0.08	-2.69	0.16	-2.22	0.13	-0.85	0.06	0.20	0.05
13	P5052R1r_PEER	65	1.60	0.10	-2.62	0.14	-2.18	0.11	-1.03	0.06	0.06	0.04
14	P733R1r_PEER	70	1.71	0.11	-3.24	0.18	-3.04	0.16	-1.88	0.09	-0.83	0.05
15	P2964R1r_PEER	75	2.27	0.16	-2.38	0.11	-2.12	0.10	-1.40	0.06	-0.67	0.04

Summed-Score Based Item Diagnostic Tables and X^2s for Group 1 (Back to TOC)

S-X^2 Item Level Diagnostic Statistics

Item	Label	X^2	d.f.	Probability
1	P5018R1r_PEER	87.16	76	0.1791
2	P5058R1r_PEER	97.20	76	0.0510
3	P5056R1r_PEER	94.69	85	0.2210
4	P1147R1r_PEER	108.29	69	0.0018
5	P5055R1r_PEER	82.43	67	0.0968
6	P233R2r_PEER	78.93	75	0.3553
7	P210R1r_PEER	82.92	66	0.0777
8	P9020R1r_PEER	82.58	67	0.0949
9	P726aR2r_PEER	69.46	65	0.3291
10	P9019r_PEER	69.17	66	0.3703
11	P5152R1r_PEER	135.96	91	0.0016
12	P5150R1r_PEER	79.56	87	0.7025
13	P5052R1r_PEER	86.48	75	0.1715
14	P733R1r_PEER	108.50	56	0.0001
15	P2964R1r_PEER	100.85	62	0.0013

Graded Model Item Parameter Estimates, logit: $a\theta + c$

Item	Label		a	s.e.		c_1	s.e.		c_2	s.e.		c_3	s.e.		c_4	s.e.
1	P5018R1r_PEER	80	3.18	0.25	76	8.89	0.68	77	7.24	0.50	78	4.47	0.31	79	0.91	0.19
2	P5058R1r_PEER	85	3.57	0.29	81	9.28	0.69	82	7.72	0.54	83	4.69	0.34	84	0.86	0.21

3	P5056R1r_PEER	90	2.91	0.22	86	7.89	0.56	87	5.61	0.36	88	2.82	0.22	89	0.14	0.17
4	P1147R1r_PEER	95	3.01	0.22	91	6.91	0.45	92	5.14	0.32	93	2.77	0.22	94	0.08	0.17
5	P5055R1r_PEER	100	4.18	0.34	96	11.82	0.99	97	9.48	0.72	98	5.42	0.41	99	0.89	0.23
6	P233R2r_PEER	105	2.81	0.20	101	6.91	0.45	102	4.75	0.30	103	1.57	0.18	104	-1.36	0.18
7	P210R1r_PEER	110	3.11	0.23	106	7.40	0.49	107	5.41	0.34	108	2.10	0.21	109	-1.66	0.20
8	P9020R1r_PEER	115	3.19	0.24	111	9.45	0.75	112	6.41	0.41	113	2.96	0.24	114	-0.77	0.18
9	P726aR2r_PEER	120	4.02	0.32	116	10.43	0.81	117	7.83	0.55	118	5.28	0.40	119	0.87	0.22
10	P9019r_PEER	125	2.88	0.22	121	7.58	0.52	122	6.08	0.39	123	3.34	0.24	124	-0.01	0.17
11	P5152R1r_PEER	130	0.88	0.11	126	3.24	0.20	127	2.14	0.14	128	0.71	0.11	129	-1.29	0.12
12	P5150R1r_PEER	135	2.21	0.17	131	6.15	0.40	132	4.29	0.26	133	2.00	0.17	134	-0.83	0.15
13	P5052R1r_PEER	140	3.40	0.26	136	8.28	0.57	137	6.32	0.41	138	3.37	0.26	139	-0.65	0.19
14	P733R1r_PEER	145	3.07	0.24	141	9.81	0.85	142	7.42	0.51	143	4.27	0.30	144	0.22	0.18
15	P2964R1r_PEER	150	4.15	0.35	146	11.69	0.99	147	9.70	0.76	148	5.80	0.44	149	1.28	0.24

Graded Model Item Parameter Estimates for Group 2, logit: $a(\theta - b)$ (Back to TOC)

Item	Label		*a*	*s.e.*	b_1	*s.e.*	b_2	*s.e.*	b_3	*s.e.*	b_4	*s.e.*
1	P5018R1r_PEER	80	3.18	0.25	-2.80	0.19	-2.28	0.13	-1.41	0.07	-0.29	0.05
2	P5058R1r_PEER	85	3.57	0.29	-2.59	0.16	-2.16	0.12	-1.31	0.07	-0.24	0.05
3	P5056R1r_PEER	90	2.91	0.22	-2.71	0.18	-1.93	0.10	-0.97	0.06	-0.05	0.06
4	P1147R1r_PEER	95	3.01	0.22	-2.30	0.13	-1.71	0.09	-0.92	0.06	-0.03	0.06
5	P5055R1r_PEER	100	4.18	0.34	-2.83	0.19	-2.27	0.12	-1.30	0.06	-0.21	0.05
6	P233R2r_PEER	105	2.81	0.20	-2.46	0.15	-1.69	0.09	-0.56	0.05	0.48	0.07
7	P210R1r_PEER	110	3.11	0.23	-2.38	0.14	-1.74	0.09	-0.68	0.05	0.53	0.07
8	P9020R1r_PEER	115	3.19	0.24	-2.96	0.21	-2.01	0.11	-0.93	0.06	0.24	0.06
9	P726aR2r_PEER	120	4.02	0.32	-2.59	0.16	-1.95	0.10	-1.31	0.06	-0.22	0.05
10	P9019r_PEER	125	2.88	0.22	-2.63	0.17	-2.11	0.12	-1.16	0.06	0.00	0.06
11	P5152R1r_PEER	130	0.88	0.11	-3.67	0.41	-2.43	0.26	-0.80	0.12	1.46	0.23
12	P5150R1r_PEER	135	2.21	0.17	-2.79	0.19	-1.95	0.11	-0.91	0.06	0.38	0.08
13	P5052R1r_PEER	140	3.40	0.26	-2.43	0.14	-1.86	0.09	-0.99	0.06	0.19	0.06
14	P733R1r_PEER	145	3.07	0.24	-3.19	0.26	-2.41	0.14	-1.39	0.07	-0.07	0.06
15	P2964R1r_PEER	150	4.15	0.35	-2.82	0.18	-2.34	0.13	-1.40	0.07	-0.31	0.05

Summed-Score Based Item Diagnostic Tables and X^2s for Group 2 (Back to TOC)

S-X^2 Item Level Diagnostic Statistics

Item	Label	X^2	*d.f.*	Probability
1	P5018R1r_PEER	52.41	51	0.4204
2	P5058R1r_PEER	59.95	48	0.1152
3	P5056R1r_PEER	75.87	57	0.0479
4	P1147R1r_PEER	59.64	65	0.6650
5	P5055R1r_PEER	46.38	42	0.2959
6	P233R2r_PEER	79.64	65	0.1044
7	P210R1r_PEER	78.07	59	0.0488
8	P9020R1r_PEER	63.48	54	0.1767
9	P726aR2r_PEER	55.73	49	0.2361
10	P9019r_PEER	70.17	57	0.1127
11	P5152R1r_PEER	214.34	107	0.0001
12	P5150R1r_PEER	85.01	71	0.1226
13	P5052R1r_PEER	51.80	57	0.6707
14	P733R1r_PEER	66.09	47	0.0344
15	P2964R1r_PEER	51.96	39	0.0799

Group Parameter Estimates (Back to TOC)

Group	Label	μ	*s.e.*	σ^2	*s.e.*	σ	*s.e.*
1	G0	0.00	-----	1.00	-----	1.00	-----
2	G1	-0.33	-----	0.79	-----	0.89	-----

DIF Statistics for Graded Items (Back to TOC)

Item numbers in:										
Group 1	Group 2	Total X^2	*d.f.*	*p*	X^2_a	*d.f.*	*p*	$X^2_{c\mid a}$	*d.f.*	*p*
1	1	90.7	5	0.0001	15.3	1	0.0001	75.4	4	0.0001
2	2	95.6	5	0.0001	30.8	1	0.0001	64.8	4	0.0001
3	3	105.8	5	0.0001	26.7	1	0.0001	79.1	4	0.0001
4	4	37.4	5	0.0001	14.2	1	0.0002	23.1	4	0.0001
5	5	81.7	5	0.0001	33.0	1	0.0001	48.7	4	0.0001
6	6	45.2	5	0.0001	11.2	1	0.0008	33.9	4	0.0001
7	7	50.0	5	0.0001	8.4	1	0.0037	41.6	4	0.0001
8	8	58.9	5	0.0001	13.4	1	0.0002	45.5	4	0.0001
9	9	89.7	5	0.0001	39.9	1	0.0001	49.8	4	0.0001
10	10	87.9	5	0.0001	15.8	1	0.0001	72.1	4	0.0001
11	11	67.1	5	0.0001	0.3	1	0.5969	66.9	4	0.0001
12	12	79.6	5	0.0001	25.1	1	0.0001	54.5	4	0.0001
13	13	81.5	5	0.0001	43.7	1	0.0001	37.8	4	0.0001
14	14	120.4	5	0.0001	26.3	1	0.0001	94.1	4	0.0001
15	15	82.6	5	0.0001	24.0	1	0.0001	58.7	4	0.0001

Marginal fit (X^2) and Standardized LD X^2 Statistics for Group 1 (Back to TOC)

Item	Label	Marginal X^2	1	2	3	4	5	6	7	8	9	10
1	P5018R1r_PEER	4.8										
2	P5058R1r_PEER	0.5	7.8									
3	P5056R1r_PEER	0.1	8.3	16.1								
4	P1147R1r_PEER	0.9	24.9	10.2	4.3							
5	P5055R1r_PEER	3.9	7.4	17.1	7.2	2.5						
6	P233R2r_PEER	0.2	14.3	12.1	3.8	10.3	11.4					
7	P210R1r_PEER	0.2	24.9	16.6	10.5	10.9	4.6	31.7				
8	P9020R1r_PEER	0.5	13.1	13.9	7.6	8.0	15.9	28.4	32.2			

9	P726aR2r_PEER	0.7	2.8	8.3	3.6	1.4	5.4	14.0	6.2	6.8		
10	P9019r_PEER	2.2	3.5	9.7	7.2	1.5	3.8	4.6	1.8	1.3	2.2	
11	P5152R1r_PEER	0.3	2.7	5.9	5.5	4.3	4.4	3.0	3.9	3.3	4.3	7.5
12	P5150R1r_PEER	2.5	2.8	4.8	2.5	2.2	10.8	3.1	2.4	7.3	6.4	1.5
13	P5052R1r_PEER	4.3	5.3	11.1	8.2	3.3	2.1	5.1	4.3	3.5	4.3	6.0
14	P733R1r_PEER	1.2	14.6	17.8	15.4	3.6	10.3	7.4	12.8	8.1	6.0	3.4
15	P2964R1r_PEER	1.5	6.6	9.2	3.5	5.0	8.7	19.7	7.0	6.2	12.1	4.8

Item	Label	Marginal χ^2	11	12	13	14
11	P5152R1r_PEER	0.3				
12	P5150R1r_PEER	2.5	5.1			
13	P5052R1r_PEER	4.3	11.6	2.2		
14	P733R1r_PEER	1.2	4.5	8.1	6.9	
15	P2964R1r_PEER	1.5	4.4	4.3	9.8	8.8

Marginal fit (χ^2) and Standardized LD χ^2 Statistics for Group 2 (Back to TOC)

Item	Label	Marginal χ^2	1	2	3	4	5	6	7	8	9	10
1	P5018R1r_PEER	1.0										
2	P5058R1r_PEER	1.4	2.9									
3	P5056R1r_PEER	0.9	0.3	9.7								
4	P1147R1r_PEER	1.0	-0.2	2.4	5.6							
5	P5055R1r_PEER	2.6	2.3	4.8	3.0	4.1						
6	P233R2r_PEER	0.9	1.8	3.7	2.6	3.8	4.1					
7	P210R1r_PEER	0.8	3.0	6.9	5.5	3.6	6.2	26.2				
8	P9020R1r_PEER	0.7	2.0	2.9	0.5	3.9	3.1	9.8	20.8			
9	P726aR2r_PEER	1.9	2.4	0.5	0.8	4.3	4.1	4.1	5.7	2.3		
10	P9019r_PEER	0.6	4.6	1.2	2.9	1.8	2.5	2.1	6.6	3.6	8.9	
11	P5152R1r_PEER	0.2	8.1	10.2	4.5	7.5	7.2	9.0	10.9	8.9	6.9	17.6
12	P5150R1r_PEER	0.4	-0.3	4.2	1.6	1.0	3.1	3.5	7.7	4.6	1.2	4.1
13	P5052R1r_PEER	1.2	1.2	1.6	4.3	1.3	1.5	10.9	4.3	3.4	3.8	2.1
14	P733R1r_PEER	1.1	1.4	0.7	2.2	6.7	3.7	12.5	13.7	1.3	3.1	4.0
15	P2964R1r_PEER	2.2	1.0	2.1	1.0	6.6	3.1	11.9	9.4	3.5	1.7	5.7

Item	Label	Marginal χ^2	11	12	13	14
11	P5152R1r_PEER	0.2				
12	P5150R1r_PEER	0.4	12.9			
13	P5052R1r_PEER	1.2	15.1	6.7		
14	P733R1r_PEER	1.1	11.0	6.6	5.8	
15	P2964R1r_PEER	2.2	11.9	6.3	3.3	11.5

Item Information Function Values for Group 1 at 15 Values of θ from -2.8 to 2.8 (Back to TOC)

Item	Label	θ: -2.8	-2.4	-2.0	-1.6	-1.2	-0.8	-0.4	-0.0	0.4	0.8	1.2	1.6	2.0	2.4	2.8
1	P5018R1r_PEER	0.64	1.03	1.31	1.38	1.38	1.37	1.32	1.13	0.78	0.43	0.21	0.09	0.04	0.02	0.01
2	P5058R1r_PEER	0.63	0.90	1.07	1.10	1.10	1.12	1.10	1.01	0.78	0.50	0.28	0.14	0.07	0.03	0.02
3	P5056R1r_PEER	0.52	0.71	0.85	0.89	0.87	0.87	0.89	0.88	0.79	0.63	0.43	0.26	0.14	0.08	0.04
4	P1147R1r_PEER	0.94	1.23	1.31	1.27	1.28	1.31	1.27	1.10	0.76	0.43	0.21	0.10	0.04	0.02	0.01
5	P5055R1r_PEER	1.01	1.29	1.33	1.29	1.32	1.34	1.29	1.07	0.70	0.37	0.18	0.08	0.03	0.02	0.01
6	P233R2r_PEER	0.73	1.06	1.24	1.24	1.20	1.22	1.22	1.20	1.09	0.81	0.49	0.25	0.12	0.05	0.02
7	P210R1r_PEER	0.97	1.47	1.68	1.57	1.47	1.57	1.61	1.59	1.39	0.91	0.45	0.20	0.08	0.03	0.01
8	P9020R1r_PEER	1.11	1.43	1.50	1.42	1.41	1.45	1.44	1.36	1.06	0.63	0.31	0.14	0.06	0.02	0.01
9	P726aR2r_PEER	0.93	1.01	1.02	1.02	1.03	0.99	0.86	0.64	0.40	0.22	0.11	0.06	0.03	0.01	0.01
10	P9019r_PEER	0.93	1.05	1.05	1.05	1.07	1.07	0.98	0.76	0.50	0.28	0.14	0.07	0.03	0.02	0.01
11	P5152R1r_PEER	0.21	0.21	0.21	0.21	0.21	0.21	0.21	0.20	0.19	0.18	0.16	0.14	0.11	0.09	0.07
12	P5150R1r_PEER	0.45	0.49	0.50	0.50	0.50	0.50	0.50	0.48	0.44	0.37	0.28	0.20	0.14	0.09	0.05
13	P5052R1r_PEER	0.68	0.76	0.78	0.77	0.77	0.77	0.76	0.72	0.62	0.46	0.31	0.18	0.10	0.06	0.03
14	P733R1r_PEER	0.86	0.86	0.87	0.87	0.86	0.80	0.65	0.46	0.28	0.16	0.08	0.04	0.02	0.01	0.01
15	P2964R1r_PEER	1.06	1.45	1.57	1.58	1.56	1.49	1.22	0.76	0.39	0.17	0.07	0.03	0.01	0.00	0.00
	Test Information:	12.67	15.96	17.28	17.15	17.02	17.07	16.33	14.36	11.16	7.55	4.72	2.98	2.04	1.55	1.30
	Expected s.e.:	0.28	0.25	0.24	0.24	0.24	0.24	0.25	0.26	0.30	0.36	0.46	0.58	0.70	0.80	0.88

Marginal Reliability for Response Pattern Scores: 0.88

Item Information Function Values for Group 2 at 15 Values of θ from -2.8 to 2.8 (Back to TOC)

Item	Label	θ: -2.8	-2.4	-2.0	-1.6	-1.2	-0.8	-0.4	-0.0	0.4	0.8	1.2	1.6	2.0	2.4	2.8
1	P5018R1r_PEER	2.80	2.99	2.67	2.69	2.51	2.14	2.57	2.07	0.92	0.30	0.09	0.03	0.01	0.00	0.00
2	P5058R1r_PEER	2.89	3.80	3.43	3.18	3.27	2.50	3.13	2.68	1.07	0.30	0.07	0.02	0.00	0.00	0.00
3	P5056R1r_PEER	2.18	2.36	2.39	2.21	2.26	2.31	2.22	2.20	1.43	0.61	0.21	0.07	0.02	0.01	0.00
4	P1147R1r_PEER	1.35	2.35	2.68	2.61	2.52	2.51	2.37	2.37	1.54	0.64	0.21	0.07	0.02	0.01	0.00
5	P5055R1r_PEER	4.74	4.85	3.73	3.56	4.32	2.73	3.95	3.61	1.16	0.25	0.05	0.01	0.00	0.00	0.00
6	P233R2r_PEER	1.60	2.18	2.24	2.16	1.87	2.02	2.10	1.96	2.08	1.64	0.82	0.31	0.11	0.04	0.01
7	P210R1r_PEER	1.64	2.61	2.79	2.57	2.19	2.51	2.26	1.95	2.43	2.05	0.96	0.33	0.10	0.03	0.01
8	P9020R1r_PEER	2.64	2.48	2.72	2.29	2.44	2.60	2.07	2.42	2.40	1.25	0.43	0.13	0.04	0.01	0.00
9	P726aR2r_PEER	3.46	4.33	4.51	4.33	4.04	2.62	3.71	3.37	1.15	0.26	0.05	0.01	0.00	0.00	0.00
10	P9019r_PEER	2.06	2.47	2.35	2.15	2.25	1.97	1.92	2.14	1.53	0.69	0.25	0.08	0.03	0.01	0.00
11	P5152R1r_PEER	0.24	0.24	0.24	0.24	0.24	0.24	0.23	0.23	0.23	0.22	0.22	0.21	0.19	0.17	0.14
12	P5150R1r_PEER	1.33	1.43	1.43	1.38	1.37	1.35	1.25	1.26	1.27	0.99	0.59	0.29	0.13	0.05	0.02
13	P5052R1r_PEER	2.02	3.21	3.36	3.00	3.00	2.80	2.13	2.77	2.57	1.15	0.35	0.09	0.02	0.01	0.00
14	P733R1r_PEER	2.59	2.61	2.26	2.42	2.31	1.74	2.07	2.36	1.45	0.57	0.18	0.05	0.02	0.00	0.00
15	P2964R1r_PEER	4.75	4.89	3.52	3.99	3.87	2.71	4.25	2.94	0.82	0.17	0.03	0.01	0.00	0.00	0.00
	Test Information:	37.56	44.07	41.60	40.04	39.73	33.99	37.50	35.58	23.32	12.36	5.79	2.97	1.96	1.60	1.46
	Expected s.e.:	0.16	0.15	0.16	0.16	0.16	0.17	0.16	0.17	0.21	0.28	0.42	0.58	0.72	0.79	0.83

Marginal Reliability for Response Pattern Scores: 0.94

Likelihood-based Values and Goodness of Fit Statistics (Back to TOC)

Statistics based on the loglikelihood	
-2loglikelihood:	78038.29
Akaike Information Criterion (AIC):	78338.29

Bayesian Information Criterion (BIC):	79348.52

Statistics based on the full item x item x ... classification
The table is too sparse to compute the general multinomial goodness of fit statistics.

Statistics based on one- and two-way marginal tables
M_2 statistics not available for this estimation method.

Summary of the Data and Control Parameters **(Back to TOC)**

Group:	Group 1	Group 2
Sample Size	5689	527
Number of Items	15	15
Number of Dimensions	1	1

Group 1

Item	Label	Categories	Model
1	P5018R1r_PEER	5	Graded
2	P5058R1r_PEER	5	Graded
3	P5056R1r_PEER	5	Graded
4	P1147R1r_PEER	5	Graded
5	P5055R1r_PEER	5	Graded
6	P233R2r_PEER	5	Graded
7	P210R1r_PEER	5	Graded
8	P9020R1r_PEER	5	Graded
9	P726aR2r_PEER	5	Graded
10	P9019r_PEER	5	Graded
11	P5152R1r_PEER	5	Graded
12	P5150R1r_PEER	5	Graded
13	P5052R1r_PEER	5	Graded
14	P733R1r_PEER	5	Graded
15	P2964R1r_PEER	5	Graded

Group 2

Item	Label	Categories	Model
1	P5018R1r_PEER	5	Graded
2	P5058R1r_PEER	5	Graded
3	P5056R1r_PEER	5	Graded
4	P1147R1r_PEER	5	Graded
5	P5055R1r_PEER	5	Graded
6	P233R2r_PEER	5	Graded
7	P210R1r_PEER	5	Graded
8	P9020R1r_PEER	5	Graded
9	P726aR2r_PEER	5	Graded
10	P9019r_PEER	5	Graded
11	P5152R1r_PEER	5	Graded
12	P5150R1r_PEER	5	Graded
13	P5052R1r_PEER	5	Graded
14	P733R1r_PEER	5	Graded
15	P2964R1r_PEER	5	Graded

Parameter Estimation Control Values

Bock-Aitkin EM Algorithm		
Maximum number of cycles:	500	
Convergence criterion:	1.00e-005	
Maximum number of M-step iterations:	50	
Convergence criterion for iterative M-steps:	1.00e-006	
Number of rectangular quadrature points:	49	
Minimum, Maximum quadrature points:	-6.00	6.00
SEM algorithm tolerance:	1.00e-003	
Standard error computation algorithm:	Supplemented EM	

DIF Analysis
All items are evaluated for DIF
(Conditional on population distribution estimates obtained with all items constrained equal)
Contrasts among groups:

Group:	1	2
Contrast 1	1.000	-1.000

Miscellaneous Control Values

Print parameter numbers?	Yes
Z tolerance, max. abs. logit value:	50.00
Number of processor cores used:	8
Number of cycles completed:	221
Maximum parameter change:	0.00e+000
Number of free parameters:	150

Processing times (in seconds)

E-step computations:	0.28
M-step computations:	0.21
Standard error computations:	1.38
Goodness-of-fit statistics:	0.08
Total:	1.95

Output Files

HTML results and control parameters:	C:\AMC Werk\IRT PRO\PeerrelDIFvoorIRTPRO-ReverseGroupCoding.Test1-irt.htm

Convergence and Numerical Stability

Engine status:	Normal termination
SEM algorithm status:	Normal
First-order test:	Convergence criteria satisfied
Condition number of information matrix:	1.58e+003
Second-order test:	Solution is a possible local maximum
